# Analysis of Patients with Ventricular Assist Devices Presenting to an Urban Emergency Department

**DOI:** 10.5811/westjem.2018.8.38851

**Published:** 2018-09-10

**Authors:** Ryan P. McKillip, Anand Gopalsami, Magdeline Montoya, Gene Kim, James J. Walter, Colleen Juricek, Eric Shappell

**Affiliations:** * Advocate Christ Medical Center, Department of Emergency Medicine, Oak Lawn, Illinois; † Cedars-Sinai Medical Center, Department of Emergency Medicine, Los Angeles, California; ‡ University of California San Francisco, Department of Pediatrics, San Francisco, California; § University of Chicago, Department of Medicine, Section of Cardiology, Chicago, Illinois; ¶ University of Chicago, Department of Medicine, Section of Emergency Medicine, Chicago, Illinois; || University of Chicago, Department of Surgery, Chicago, Illinois; # Harvard University, Massachusetts General Hospital, Department of Emergency Medicine, Boston, Massachusetts

## Abstract

**Introduction:**

Left ventricular assist device (LVAD) insertion is an increasingly common intervention for patients with advanced heart failure; however, published literature on the emergency department (ED) presentation of this population is limited. The objective of this study was to characterize ED presentations of patients with LVADs with a focus on device-specific complications to inform provider education and preparation initiatives.

**Methods:**

This was a retrospective chart review of all patients with LVADs followed at an urban academic medical center presenting to the ED over a five-year period (July 1, 2009, to June 30, 2014). Two abstractors reviewed 45 randomly selected charts to standardize the abstraction process and establish a priori categories for reason for presentation to the ED. Remaining charts were then divided evenly for review by one of the two abstractors. Primary outcomes for this study were (1) frequency of and (2) reason for presentation to the ED by patients with LVADs.

**Results:**

Of 349 patients with LVADs identified, 143 (41.0%) had ED encounters during the study period. There were 620 total ED encounters, (range 1 to 32 encounters per patient, median=3, standard deviation=5.3). Among the encounters, 431 (69.5%) resulted in admission. The most common reasons for presentation were bleeding (e.g., gastrointestinal, epistaxis) (182, 29.4%); infection (127, 20.5%); heart failure exacerbation (68, 11.0%); pain (56, 9.0%); other (45, 7.3%); and arrhythmias (40, 6.5%). Fifty-two encounters (8.4%) were device-specific; these patients frequently presented with abnormal device readings (37, 6.0%). Interventions for device-specific presentations included anticoagulation regimen adjustment (16/52, 30.8%), pump exchange (9, 17.3%), and hardware repair (6, 11.5%). Pump thrombosis occurred in 23 cases (3.7% of all encounters). No patients required cardiopulmonary resuscitation or died in the ED.

**Conclusion:**

This is the largest study known to the investigators to report the rate of ED presentations of patients with LVADs and provide analysis of device-specific presentations. In patients who do have device-specific ED presentations, pump thrombosis is a common diagnosis and can present without device alarms. Specialized LVAD education and preparation initiatives for ED providers should emphasize the recognition and management of the most common and critical conditions for this patient population, which have been identified in this study as bleeding, infection, heart failure, and pump thrombosis.

## INTRODUCTION

With over 10,000 implantations to date, left ventricular assist device (LVAD) insertion as a bridge-to-transplant, bridge-to-recovery, or destination therapy, is an increasingly common intervention for patients with advanced heart failure,^1^,^2^ yet most emergency physicians have limited training or experience in the care of such patients. Numerous clinical studies have illustrated the effectiveness and complications of LVADs,^3^,^4^ but literature on the emergency department (ED) presentation of this population is limited, particularly with regard to device-specific complications.^5^–^7^ In addition to the complications associated with heart failure, patients with LVADs are at risk for critical adverse events such as intracranial and gastrointestinal bleeding, driveline infection, and pump thrombosis. Early diagnosis of pump thrombosis is critical, as it can result in urgent transplantation, device replacement, or death. Incidence has been reported as 0.02 to 0.08 events per patient per year with continuous-flow devices.^8^

While investigators have proposed pathways for evaluating patients with LVADs and assessing device function in the ED,^9^–^11^ the incidence and nature of ED encounters in this patient population remains unclear. Increased awareness regarding the common ED presentations of patients with LVADs could lead to more targeted education interventions, improved provider preparedness, and enhanced care for this complicated population. The purpose of this study was to characterize the presentation and clinical course of patients with LVADs presenting to an urban, academic medical center ED with a focus on device-specific complications.

## METHODS

### Study Design

This was a retrospective chart review of ED visits made by patients with LVADs during a five-year period (July 1, 2009 – June 30, 2014). The institutional review board approved the study protocol and waived informed consent requirements.

### Study Setting and Population

The study site was an urban, academic medical center with approximately 60,000 annual adult ED visits. The institution’s heart failure service maintains a database of all patients who have received LVADs at the institution. We queried a health record database for ED encounters by all 349 patients who had LVADs during the study period. Encounters that occurred prior to a patient’s LVAD placement or after heart transplant were excluded.

### Study Protocol and Measurements

Abstraction of the chart data used a combined deductive and inductive process. Data extracted for each encounter included patient demographics, chief complaint, evaluation, diagnostic testing, interventions, final ED ICD-9 diagnoses, and disposition. Two physician authors (ES, AG) reviewed 45 randomly selected encounters to develop presentation categories: device-specific; bleeding (e.g. gastrointestinal [GI], epistaxis); infection (e.g. bacteremia, driveline infection); heart failure exacerbation; arrhythmia; anemia; pain (chest, abdominal, or other); neurologic; dehydration; musculoskeletal; pulmonary; GI (non-bleeding); venous-access related; or other (including endocrine, renal, rheumatologic, oncologic, dermatologic, or psychiatric presentations).

We subcategorized device-specific presentations as abnormal device readings/alarms, grossly damaged equipment, or non-specific complaints. Bleeding and driveline infections, while related to having an LVAD due to requisite anticoagulation and percutaneous wiring, respectively, were not categorized as device-specific. Presentation categories were determined after review of the entire chart and were not mutually exclusive (e.g., a patient presenting with dyspnea who is diagnosed with a heart failure exacerbation from pump thrombosis would be categorized as both “heart failure exacerbation” and “device-specific: non-specific complaint”).

The abstractors used the 14 a priori presentation categories and three subcategories to sort the remaining encounters. Conflicting or ambiguous chart elements were discussed between abstractors until consensus interpretation was reached. Interrater percent agreement on 10 random charts was calculated (satisfactory agreement >=90%). As a secondary analysis, we studied outcomes in bounce-back encounters (defined as a second ED visit within seven days of discharge).

### Data Analysis

We analyzed data to calculate the frequency of, and reason for, presentation to the ED by patients with LVADs. A detailed review of device-specific encounters was performed to better understand the disposition and interventions in these patients. We compared categorical and continuous data using chi-squared and single-tailed unpaired t-testing, respectively.

## RESULTS

Of the 349 patients with LVADs during the study period, there were 838 total encounters by 158 patients. Of these, 620 encounters made by 143 patients with LVADs (116 HeartMate II™, 27 HeartWare™) met inclusion criteria. The median number of encounters made by each patient was three (range 1–32, standard deviation [SD]=5.3). Patients were mostly male (109, 76.2%), with a median age of 60 (SD=13.2) at time of first encounter. Among the encounters, 431 (69.5%) resulted in admission, 187 (30.2%) resulted in discharge, one patient left against medical advice, and one left without being seen. Interrater agreement was 100% on primary categories and 90% when secondary categories were included. The most common category was bleeding, occurring 182 (29.4%) times. Of these, 104 (104/182, 57.1%) were GI bleeding, and 57 (57/182, 31.3%) were epistaxis. Average international normalized ratio (INR) for these patients was 2.3 (N=162, SD=1.5), compared to 2.1 (N=352, SD=1.0) in other encounters in which INR was measured (P=0.08). Other common categories included 127 (20.5%) infections, 68 (11.0%) heart failure exacerbations, 56 (9.0%) pain, 45 (7.3%) other, and 40 (6.5%) arrhythmias (Figure).

No patients required cardiopulmonary resuscitation or died in the ED. Compared to other encounters, it was less common for bounce-back encounters to be device-specific (7/161 [4.3%] vs. 45/459[9.8%], P<0.01), and more common to be related to pain (25/161 [15.5%] vs. 31/459 [6.8%], P=0.02).

### Device-Specific Encounters

Fifty-two encounters (8.4%) were device-specific (Table). In the majority of these encounters, patients presented with abnormal device readings/alarms (37, 6.0% of all encounters). Patients with device-specific presentations were admitted 44 times, with seven discharges. One patient left against medical advice. Pump thrombosis occurred in 23 cases and presented with an abnormal device reading/alarm (10) or a non-specific complaint such as hematuria (6), dyspnea (3), abnormal lab value (3), or chest pain (1). Average initial INR in patients with pump thrombosis was 1.9 (N=18, SD=0.6) compared to 2.2 (N=496, SD=1.2) when measured in other encounters (P=0.17). Average lactate dehydrogenase in patients with pump thrombosis was 2142 (N=14, SD=989) compared to 451 (N=188, SD=347) when measured in other encounters (P<0.001). Interventions for device-specific presentations included anticoagulation regimen adjustment (16, 30.8%), pump exchange (9, 17.3%), hardware repair (6, 11.5%), and device settings adjustment (4, 7.7%).

## DISCUSSION

Specialized LVAD education and preparation initiatives for ED providers should focus on the most common and most critical presentations in this population. This study provides an evidentiary basis for such interventions by characterizing the frequency and nature of ED encounters for patients with LVADs. GI hemorrhage and epistaxis made bleeding the most common reason for presentation to the ED in our study, accounting for more than one in four visits. This is congruent with the results of previous studies.^6^,^7^ Risk factors for bleeding in this population include anticoagulation, development of arteriovenous malformations, and acquired von Willebrand disease.^2^ It is, therefore, extremely important that ED providers be familiar with the workup and management of bleeding complications in this population.

Infection was the second most common presentation category in our study, often presenting as bacteremia associated with a driveline infection. This is consistent with the known high risk of infection in this population, including the risk of sepsis developing in as many as 20% of patients within one year of device implantation.^1^ Heart failure exacerbations ranked third in prevalence. The importance of familiarity with the management of these conditions in this population is underscored by the frequency of these presentations.

We identified device-specific presentations in 8.4% of ED visits. Although the majority of these encounters presented with an abnormal device reading/alarm, more than one in four had normal device readings. About half of the device-specific presentations were due to pump thrombosis. Thrombosis should be suspected in cases of abnormal device readings (e.g., increased power, increased calculated flow), worsening heart failure, and hemolysis, often in the setting of subtherapeutic anticoagulation.^8^ Importantly, in our study, patients with pump thrombosis more often presented with a non-specific complaint than an abnormal device reading or alarm. Approximately one in 50 patients who presented with a non-specific complaint such as hematuria or dyspnea ultimately were diagnosed with pump thrombosis after admission for further testing. These data highlight the importance of vigilance in pursuing this diagnosis in patients with LVADs presenting to the ED.

## LIMITATIONS

This was a retrospective chart review and used subjective interpretation of medical records to develop presentation categories. By using presentation categories, our intention was to provide more meaningful information than what is typically derived from the chief complaint, final diagnosis, or other objective outputs from health records. Our investigation of interventions was limited to device-specific encounters. Therefore, we did not report data on interventions for more common presentations such as bleeding and infection. Although we studied a large sample of patients across several years, we were limited to ED presentations at a single institution, and exclusively studied patients who had their LVAD placed at that same institution. Additionally, all patients received either the HeartMate II™ or HeartWare™ device, and thus our study does not include presentations of patients with other devices.

## CONCLUSION

This is the largest study known to the investigators to report the rate of ED presentations of patients with LVADs and provide analysis of device-specific presentations. In patients that do have device-specific ED presentations, pump thrombosis is a common diagnosis and can present without device alarms. Specialized LVAD education and preparation initiatives for ED providers should emphasize the recognition and management of the most common and critical conditions for this patient population, which have been identified as bleeding, infection, heart failure, and pump thrombosis.

## Figures and Tables

**Figure:**
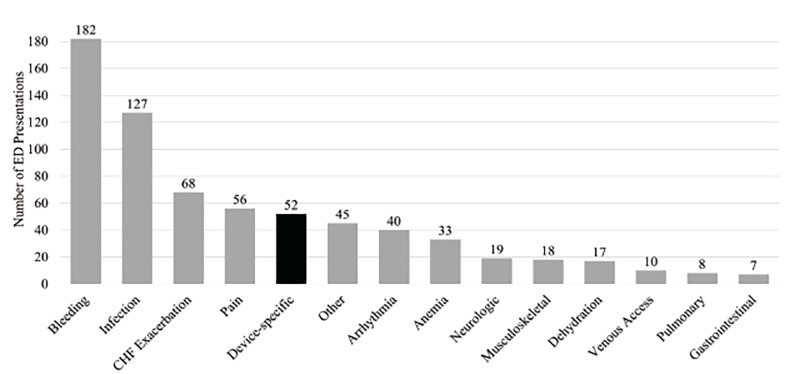
Number of emergency department (ED) presentations by category from patients with left ventricular assist devices in a five-year period. Device-specific presentations are highlighted in black. *CHF*, congestive heart failure.

**Table:** Summary of device-specific encounters and interventions.

	N (%)
Number of device-specific encounters	52
Number of unique patients	32
Device type	
HeartMate II™	23 (71.9%)
HeartWare™	9 (28.1%)
Disposition	
Admit	44 (84.7%)
Discharge	7 (13.4%)
Against medical advice	1 (1.9%)
Presentation subcategory	
Abnormal device reading/alarm	37 (71.2%)
Grossly damaged equipment	2 (3.8%)
Non-specific complaint	13 (25.0%)
Interventions for device-specific encounters	
Anticoagulation adjustment	16 (30.8%)
Pump exchange	9 (17.3%)
Hardware repair, replacement, or adjustment	6 (11.5%)
Device settings adjustment	4 (7.7%)
Catheter-directed thrombolysis	4 (7.7%)
Heart transplant	2 (3.8%)
Diuresis	2 (3.8%)
Other	7 (13.5%)
No intervention	3 (5.8%)

## References

[1] Prinzing A, Herold U, Berkefeld A (2016). Left ventricular assist devices—current state and perspectives. J Thorac Dis.

[2] Sajgalik P, Grupper A, Edwards BS (2016). Current status of left ventricular assist device therapy. Mayo Clin Proc.

[3] Slaughter MS, Rogers JG, Milano CA (2009). Advanced heart failure treated with continuous-flow left ventricular assist device. N Engl J Med.

[4] Rogers JG, Aaronson KD, Boyle AJ (2010). Continuous flow left ventricular assist device improves functional capacity and quality of life of advanced heart failure patients. J Am Coll Cardiol.

[5] Goebel M, Donofrio J, Serra J (2017). 68 An urban fire department’s experience with left ventricular assist devices. Ann Emerg Med.

[6] Devine A, Knapp B, Lo B (2012). 404 Characteristics and frequency of emergency department visits of patients with continuous flow left ventricular assist devices. Ann Emerg Med.

[7] Tainter C, Braun O, Teran F (2017). Emergency department visits among patients with left ventricular assist devices. Intern Emerg Med.

[8] Birati EY, Rame JE (2015). Diagnosis and management of LVAD thrombosis. Curr Treat Options Cardiovasc Med.

[9] Horton SC, Khodaverdian R, Powers A (2004). Left ventricular assist device malfunction: a systematic approach to diagnosis. J Am Coll Cardiol.

[10] Sen A, Larson JS, Kashani KB (2016). Mechanical circulatory assist devices: a primer for critical care and emergency physicians. Crit Care.

[11] Aggarwal A, Kurien S, Coyle L (2013). Evaluation and management of emergencies in patients with mechanical circulatory support devices. Prog Transplant.

